# Association of Hospital Telestroke Adoption With Changes in Initial Hospital Presentation and Transfers Among Patients With Stroke and Transient Ischemic Attacks

**DOI:** 10.1001/jamanetworkopen.2021.26612

**Published:** 2021-09-23

**Authors:** Kori S. Zachrison, Jessica V. Richard, Andrew Wilcock, Jose R. Zubizaretta, Lee H. Schwamm, Lori Uscher-Pines, Ateev Mehrotra

**Affiliations:** 1 Department of Emergency Medicine, Massachusetts General Hospital, Boston; 2 Department of Health Care Policy, Harvard Medical School, Boston, Massachusetts; 3 Department of Family Medicine, University of Vermont College of Medicine, Burlington; 4 Department of Neurology, Massachusetts General Hospital, Boston; 5 RAND Corporation, Arlington, Virginia; 6 Division of General Medicine, Beth Israel Deaconess Medical Center, Boston, Massachusetts

## Abstract

**Importance:**

It has been proposed that the implementation of telestroke services (a web-based approach to using video telecommunication to treat patients with stroke before hospital admission) changes where patients with stroke symptoms receive care, but this proposal has not been rigorously assessed.

**Objective:**

To assess whether the implementation of telestroke services is associated with changes in where and how patients initially present with stroke symptoms, in their decision to be transferred to another hospital, and which hospitals they are transferred to.

**Design, Setting, and Participants:**

This cross-sectional study compared changes in stroke systems of care between a sample of 593 US hospitals that adopted telestroke during the period from 2009 to 2016 but were not comprehensive stroke centers, major teaching hospitals, or thrombectomy-capable hospitals vs 593 matched control hospitals without telestroke based on rural location, critical access hospital status, bed size, primary stroke center status, presence of hospital alternatives in the community, hospital stroke volume, census region, and ownership. With the use of data on 100% of Medicare fee-for-service beneficiaries, all stroke and transient ischemic attack admissions from 2008 to 2018 were identified.

**Exposures:**

For each hospital pair (telestroke plus matched control), the telestroke hospital’s implementation date and difference-in-differences approach were used to quantify the association between telestroke implementation and changes in care from 2 years before implementation to 2 years after implementation. Models also controlled for differences in observed patient characteristics.

**Main Outcomes and Measures:**

Hospital stroke volume, patients’ ambulance transport distance to initial hospital, hospital case mix, interhospital transfer proportion, and size of the receiving hospital for transferred patients.

**Results:**

Of the 669 telestroke hospitals and 2143 potential control hospitals, 593 hospital pairs were matched; in each category, 261 hospitals (44.0%) were located in a rural area, 179 (30.2%) were primary stroke centers, and 130 (21.9%) were critical access hospitals. The changes in the preimplementation to postimplementation period were similar at telestroke and control hospitals in mean annual stroke volume (telestroke hospitals, decreased from 79.6 to 76.3 patients; control hospitals, decreased from 78.8 to 75.5 patients [–3.3 patients per year for both; difference-in-differences, 0.009; *P* ≥ .99]). Similarly, no differences were seen in ambulance transport distance, case mix, interhospital transfers, or bed size of receiving hospitals among transferred patients.

**Conclusions and Relevance:**

This study suggests that, across a national sample of hospitals implementing telestroke, no association between telestroke adoption and changes in stroke systems of care were found.

## Key Points

**Question:**

Was the implementation of telestroke services at hospitals associated with changes in the organization of stroke systems of care?

**Findings:**

In this cross-sectional study using a national sample of 593 telestroke hospitals and 593 matched control hospitals, no significant association was found between telestroke implementation and a hospital’s stroke volume, rate of transfers, or the hospitals to which patients are transferred.

**Meaning:**

This study suggests that a hospital’s implementation of telestroke was not associated with changes in where patients present, in their decision to transfer, or which hospital they are transferred to.

## Teaser

This cross-sectional study assesses whether the implementation of telestroke services is associated with changes in where and how patients initially present with stroke symptoms, in their decision to be transferred to another hospital, and which hospitals they are transferred to.

## Introduction

There is wide variation across hospitals in the treatment and outcomes of patients with stroke.^1,2,3,4,5,6,7,8^ In part, this variation is associated with hospital resource availability, including stroke neurologists, dedicated stroke units, neurointensive care units, and endovascular therapies.^9^ As a result, there has been a push to create multifaceted stroke systems of care that include a range of clinicians who treat the patient from the time the stroke systems begin through rehabilitation after hospital discharge. In the acute setting, systems of care recommendations include clear guidelines for prehospital triage and routing and, when necessary, transfer to hospitals with more resources.^10,11^ The underlying philosophy has been bringing the patient to the resources, and over time, interhospital transfers of patients with stroke have increased.^12,13^

For some hospitals, a key component of improving stroke systems of care is telestroke (a web-based approach to using video telecommunication to treat patients with stroke before hospital admission). Telestroke expedites the evaluation of patients by a stroke specialist and can increase the use of time-sensitive stroke interventions.^14,15^ It may also improve stroke systems of care at the adopting hospital and surrounding community.^11,16,17,18^ For example, it may change where patients initially present because emergency medical services (EMSs) introduce new transport protocols or informally as EMS professionals decide to take a patient to a hospital with telestroke because of their familiarity with stroke-related resources at local hospitals. Such a shift would be reflected in increased stroke volume, decreased distance traveled by ambulance, and hospitals’ stroke case mix. A hospital’s adoption of telestroke could also reduce the need for transfer because patients requiring evaluation by a stroke specialist may no longer need to be transferred. In addition, if the patient is transferred, the transfer destination may change; for example, an emergency department (ED) connected to an academic telestroke hub hospital may transfer patients with stroke to this hub more consistently.

Although such changes have been hypothesized, to our knowledge, the association between telestroke adoption and these measures of stroke systems of care has not been quantified across telestroke networks, owing, in part, to the challenges associated with identifying which hospitals have implemented telestroke. Our objective was to assess whether the introduction of telestroke across a national sample of hospitals was associated with changes in specific components of stroke systems of care (ie, initial hospital of presentation, hospital stroke volume, patients’ ambulance transport distance, hospital case mix, proportion of patients with interhospital transfer, and size of the receiving hospital for transferred patients).

## Methods

### Overview

We used a difference-in-differences approach to compare changes in outcomes associated with stroke systems of care among a sample of US hospitals adopting telestroke vs matched control hospitals. This design allowed us to control for temporal trends by comparing the differential changes in outcomes between telestroke hospitals and matched control hospitals from a preimplementation to postimplementation period. An underlying assumption is that the experience of the control hospitals over time describes what would have happened with a given outcome at the telestroke hospitals in the absence of telestroke implementation. This study was approved by the institutional review board of Harvard Medical School; informed consent was not required as this was a secondary use of administrative data. This study follows the Strengthening the Reporting of Observational Studies in Epidemiology (STROBE) reporting guideline for observational studies.

### Study Population

Using a 100% sample of Inpatient and Outpatient Standard Analytic Files from 2008 to 2018 for beneficiaries with traditional Medicare, we used *International Classification of Diseases, Ninth Revision* (*ICD-9*) and *International Statistical Classification of Diseases and Related Health Problems, Tenth Revision* (*ICD-10*) primary diagnosis codes to identify all stroke and transient ischemic attack (TIA) admissions from 2008 to 2018 (*ICD-9* codes 433.x [except 433.10], 434.x, 435.x, and 436; *ICD-10* codes 163.x, 164.x, 166.x, 168.89, and G45.x). When a patient had multiple contiguous claims for stroke at a single hospital or across different hospitals (eg, when transferred), the claims were combined into a single episode.

We classified episodes based on the first hospital or ED at which the patient received care. For example, if a patient was transferred, the patient would be attributed to the initial hospital of presentation. Patient characteristics from the Master Beneficiary Summary Files were demographic characteristics (age, sex, race and ethnicity, and residential zip code), Medicare enrollment (original entitlement reason and Medicaid dual eligibility), and comorbid conditions (atrial fibrillation, myocardial infarction, ischemic heart disease, diabetes, hyperlipidemia, and prior stroke or TIA).

### Hospital Sample

**Figure 1.**
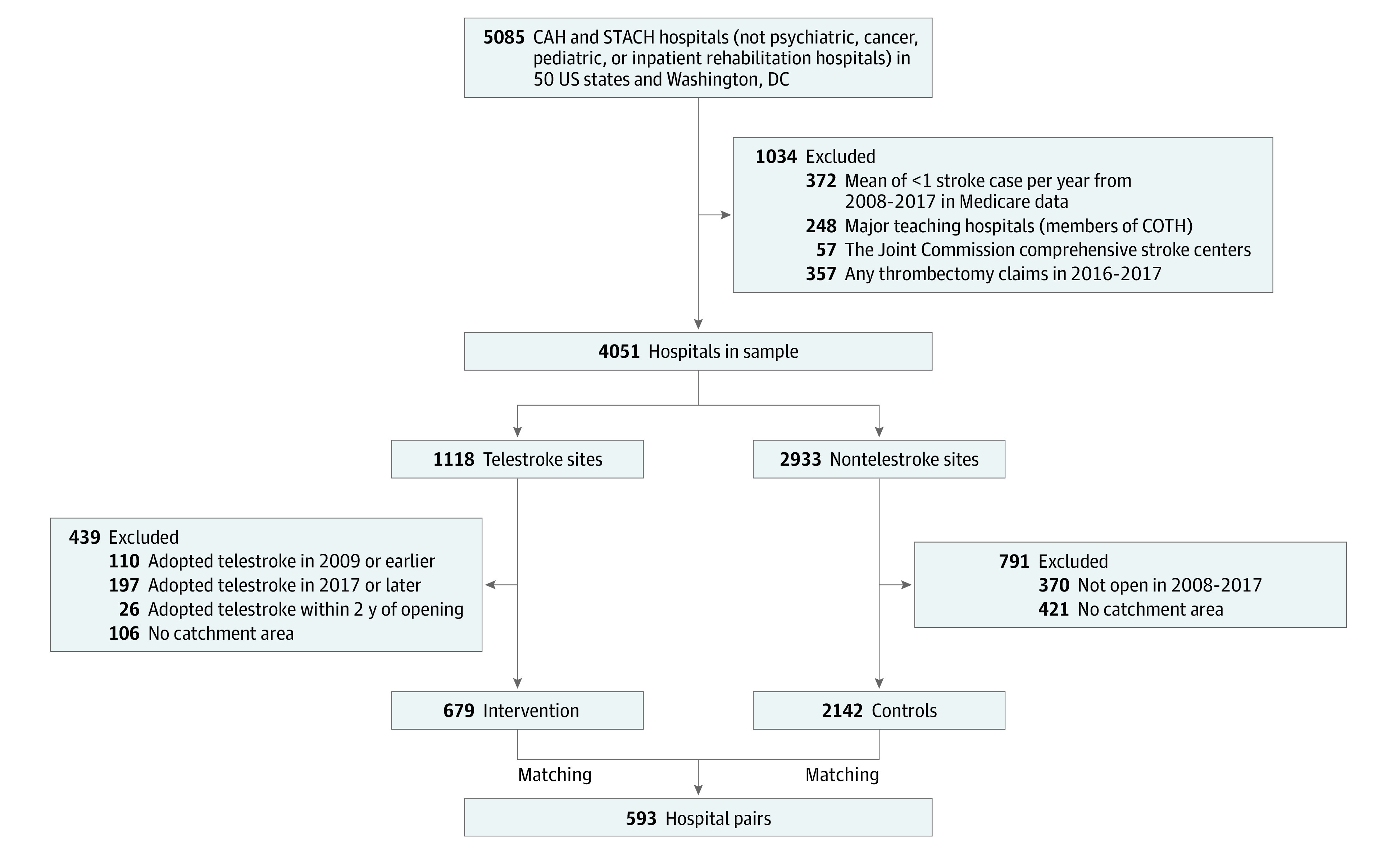
Hospital Inclusion Flow Diagram CAH indicates critical access hospital; COTH, Council of Teaching Hospitals; and STACH, short-term acute care hospital.

To focus on hospitals where we expected telestroke implementation to have the greatest association with changes in stroke systems, we excluded tertiary hospitals (ie, major teaching hospitals, thrombectomy-capable hospitals, and comprehensive stroke centers) (Figure 1). Implementation of telestroke at a tertiary hospital may be important (eg, allowing existing consultants to provide stroke consultations remotely overnight) but likely does not bring new expertise; thus, we did not expect telestroke at a tertiary hospital to be associated with changes in patterns of patient allocation or systems of care for the surrounding community. Major teaching hospitals were identified as such if they were members of the Council of Teaching Hospitals. Thrombectomy-capable hospitals were those with at least 1 thrombectomy claim during the period from 2016 to 2017 (dichotomous), and stroke center status was by The Joint Commission, Healthcare Facilities Accreditation Program, Det Norske Veritas, or Center for Improvement in Healthcare Quality criteria (comprehensive, primary stroke center [PSC], or none). We also excluded hospitals with less than 1 stroke case per year, on average, and those that implemented telestroke prior to 2009, after 2016, or within 2 years of the hospital’s opening, as we used data from 2 years before and after telestroke implementation.

### Hospital Characteristics

Our strategy for identifying telestroke hospitals has been previously described.^19^ In brief, we conducted a literature review and consultations with stroke experts to identify existing US telestroke programs. Fifteen hub-and-spoke academic networks and commercial telestroke companies provided us with the list of hospitals in their telestroke system and the month and year of initiation of the telestroke program. All other hospitals were potential control hospitals. Other characteristics captured were census region, urban or rural status, bed count, hospital type (critical access vs short-term acute-care hospital), public vs private ownership, 2008 annual stroke volume, and PSC status.

Telestroke implementation may have a differential association with hospitals with few or many alternative hospitals nearby. We identified all of the hospitals within 96 km (60 miles) of a given hospital to create a measure of alternatives by counting the number of alternatives weighted by their distance using inverse distance weighting (ie, more weight given to closer hospitals).^20^ Hospitals were categorized into terciles based on this measure of hospital alternatives (fewest, few, or more options).

### Outcomes

For each hospital, we measured the annual total stroke volume and the annual stroke volume among patients living in the catchment area. The hospital catchment area is the geographical area surrounding a hospital from which each hospital was the most common provider of stroke care. To create these areas, we used residential 5-digit zip codes for all patients with stroke during the preimplementation period (2008-2010). If most patients from a given zip code presented to 1 hospital for their initial care, then that zip code was assigned to that hospital’s catchment area. A given hospital’s catchment area was composed of zip codes in which most patients presented to that hospital. If no single hospital captured a majority of patients with stroke from a zip code, then the zip code was not assigned. Among the 1 071 186 episodes of stroke, 714 124 (66.7%) were in a zip code assigned to a hospital’s catchment area.

In a community without stroke expertise at the local hospital, the introduction of telestroke may increase access to stroke expertise closer to home. To assess whether patients could be evaluated closer to their home, we measured the mean distance traveled by an ambulance to a hospital. Ambulance data were from the carrier (Part B) claim file, which we had for a 20% random sample of all Medicare beneficiaries. We used Healthcare Common Procedure Coding System modifier code RH for residence-to-hospital transport and code SH for scene-to-hospital transport.

To examine whether telestroke implementation was associated with changes in hospital patient case mix, we measured each patient’s 180-day mortality (used in prior work^21^) based on patient age, sex, race and ethnicity, Medicaid enrollment, original Medicare entitlement reason, and indicators for 27 comorbid conditions (eMethods 1 in the Supplement).

We measured proportion of transfers as the proportion of all patients with stroke presenting to the hospital who were subsequently transferred to another hospital. Transfers were identified based on having adjacent ED or inpatient billing from 2 different hospitals on the same or consecutive days.^22,23^ Among transferred patients, we examined changes in receiving hospital bed count. Finally, we examined the discharging hospital bed count among all patients regardless of whether the patient was transferred.

### Matching

To address both differences in hospitals and in their patient populations as well as the varying dates of telestroke adoption, we used risk set and cardinality matching to match telestroke and control hospitals.^24,25^ Each telestroke hospital was matched 1:1 to a control hospital using exact matching criteria (rural or urban location, critical access hospital status, bed size [terciles], PSC status, and hospital alternatives [terciles]) and mean-balance criteria (2008 stroke volume [terciles], census region, and hospital ownership [public vs private]). We used exact matching on the characteristics associated with stroke systems of care on which we thought it would be most important to ensure perfect balance between telestroke and control hospitals. Mean-balance matching ensured that the standardized difference in sample mean values between the telestroke and control hospitals was less than the commonly accepted threshold of 0.1 absolute SDs.

We used the date of telestroke implementation to anchor each matched pair of telestroke and control hospitals together to a period in time, which we defined as the half-year in which telestroke was adopted, setting this 6-month period as time 0 for each pair. Once time 0 was defined, we looked back 2 years to characterize the preimplementation period, and we looked forward 2 years to characterize the postimplementation period. The postimplementation period began after the 6-month period during which telestroke was introduced to allow for hospitals and systems to reequilibrate before evaluating for potential change in outcomes. For example, if hospital C implemented telestroke in January 2015, we set time 0 as January to June of 2015 for both hospital C and its matched control hospital. We then identified January 2013 to December 2014 as the preimplementation period and July 2015 to June 2017 as the postimplementation period for both.

### Statistical Analysis

#### Difference-in-Differences Analysis

To estimate the association of telestroke implementation with outcomes while accounting for secular trends in stroke care, we measured the differential change in outcomes at telestroke hospitals relative to changes at control hospitals using 2 years of preimplementation data and 2 years of postimplementation data. We used a generalized linear model for each outcome of interest, accounting for clustering by matched telestroke and control hospital pairs (eMethods 2 in the Supplement). The primary effect of interest is given by whether the association between time (before vs after implementation) and the outcome varies by telestroke status.^26^

In the models examining annual total stroke volume and annual stroke volume among patients living in a hospital’s catchment area, the unit of analysis was the hospital. In the models for all other outcomes, the unit of analysis was the stroke episode. To address the potential for differential temporal changes in patient population at telestroke and control hospitals, we also included the following variables in the regression models: patient age, sex, race, ethnicity, Medicaid dual enrollment, weekend arrival at hospital, and patient medical history variables (history of atrial fibrillation, prior myocardial infarction, ischemic heart disease, diabetes, hyperlipidemia, and prior stroke or TIA). Because the 180-day estimated mortality outcome was based on patient demographic characteristics and comorbid conditions, these variables were not included in the model for that outcome. As 1 test of the assumptions in the difference-in-difference analysis, we tested for differences in preimplementation trends between telestroke and control hospitals, and they were similar (eMethods 3 and eTable 1 in the Supplement).

All hypothesis testing was 2-sided, and results were deemed significant at *P* < .05. Analyses were performed in SAS, version 9.4 (SAS Institute Inc).

#### Sensitivity Analyses

The association between telestroke adoption and stroke systems of care may vary by hospital type. Thus, we also stratified our analyses by whether a hospital was a PSC.

To address the concern that we may have included telestroke hospitals in our control group if their networks did not provide us with participants, in a second sensitivity analysis, we used data from the National Emergency Department Inventory survey on non-PSCs to identify EDs that indicated that they did not have telestroke (EDs responded to “Does your ED receive telemedicine services for patient evaluation from other facilities? Yes/No,” and if the answer was yes, EDs selecting “Stroke/Neuro” as a clinical application were considered to have telestroke).^27^ Among non-PSCs, we limited the pool of potential control hospitals to those that said no in 2016 to 2017, rematched our telestroke and control hospitals, and repeated our difference-in-differences analyses.

## Results

### Hospital Cohort

Of the 5085 hospitals in the US, we identified 4051 nontertiary hospitals that treated, on average, 1 or more patients with stroke per year from 2008 to 2018 (1118 telestroke hospitals and 2933 nontelestroke telestroke). After eliminating hospitals adopting telestroke outside of the designated time window and those without a catchment area, we had 669 telestroke sites and 2143 potential controls. We were able to match 593 hospital pairs (Figure 1) (76 pairs could not be matched because there was no available control with similar characteristics).

Before matching, compared with potential controls, telestroke hospitals were more often PSCs, in urban areas, had a higher hospital bed count, and a higher annual stroke volume (Table 1). After matching, hospitals were similar across all characteristics. Among the matched telestroke and control hospitals, 261 (44.0%) were located in a rural area, 179 (30.2%) were PSCs, and 130 (21.9%) were critical access hospitals.

**Table 1.** Hospital Characteristics Overall and by Telestroke Status, Presented at the Hospital Level

Characteristic	Before matching			After matching		
	By telestroke status, No. (%)		*P* value	By telestroke status, No. (%)		*P* value
	Telestroke hospitals (n = 669)	Nontelestroke hospitals (n = 2143)		Telestroke hospitals (n = 593)	Control hospitals (n = 593)	
Primary stroke center^a^						
Yes	218 (32.6)	409 (19.1)	<.001	179 (30.2)	179 (30.2)	>.99
No	451 (67.4)	1734 (80.9)		414 (69.8)	414 (69.8)	
Urban vs rural^a^						
Urban	367 (54.9)	900 (42.0)	<.001	332 (56.0)	332 (56.0)	>.99
Rural	302 (45.1)	1243 (58.0)		261 (44.0)	261 (44.0)	
No. of beds (terciles)^a^						
<30 (T1)	124 (18.5)	830 (38.7)	<.001	122 (20.6)	122 (20.6)	>.99
30-142 (T2)	238 (35.6)	690 (32.2)		204 (34.4)	204 (34.4)	
≥143 (T3)	307 (45.9)	623 (29.1)		267 (45.0)	267 (45.0)	
Hospital alternatives (terciles)^a^						
Fewest options (T1)	183 (27.4)	775 (36.2)	<.001	161 (27.2)	161 (27.2)	>.99
Few options (T2)	238 (35.6)	746 (34.8)		198 (33.4)	198 (33.4)	
More options (T3)	248 (37.1)	622 (29.0)		234 (39.5)	234 (39.5)	
Hospital type^a^						
STACH	526 (78.6)	1264 (59.0)	<.001	463 (78.1)	463 (78.1)	>.99
CAH	143 (21.4)	879 (41.0)		130 (21.9)	130 (21.9)	
Census region of hospital						
1 (Northeast)	71 (10.6)	247 (11.5)	<.001	63 (10.6)	69 (11.6)	.81
2 (Midwest)	163 (24.4)	845 (39.4)		148 (25.0)	149 (25.1)	
3 (South)	270 (40.4)	720 (33.6)		240 (40.5)	246 (41.5)	
4 (West)	165 (24.7)	331 (15.4)		142 (23.9)	129 (21.8)	
Ownership						
Public	132 (19.7)	640 (29.9)	<.001	121 (20.4)	118 (19.9)	.46
Private	537 (80.3)	1503 (70.1)		472 (79.6)	475 (80.1)	
Annual stroke volume, 2008 (terciles)						
<30 (T1)	124 (18.5)	835 (39.0)	<.001	119 (20.1)	117 (19.7)	.89
30-76 (T2)	238 (35.6)	696 (32.5)		209 (35.2)	217 (36.6)	
≥77 (T3)	307 (45.9)	612 (28.6)		265 (44.7)	259 (43.7)	

Abbreviations: CAH, critical access hospital; STACH, short-term acute care hospital; T1, tercile 1; T2, tercile 2; T3, tercile 3.

^a^ Variables on which hospitals were exact matched. Variables that were not exact matched were mean-balanced.

### Main Results

**Figure 2.**
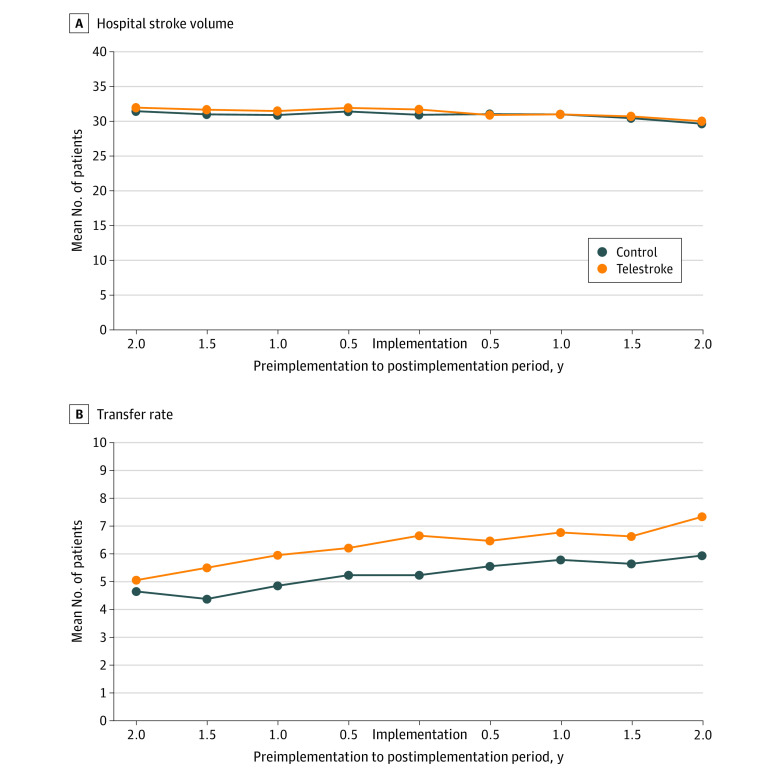
Trends in Select Outcomes Between Telestroke and Matched Control Hospitals

Telestroke and control hospitals had similar changes in mean annual stroke volume (Table 2; Figure 2) from the preimplementation to postimplementation period. The mean stroke volume decreased from 79.6 to 76.3 patients per year (change, −3.3 per year) at telestroke hospitals and decreased from 78.8 to 75.5 patients per year (change, −3.3 per year) at control hospitals, with an estimated differential change of 0.009 (*P* > .99). The volume of patients with stroke from within the catchment area also decreased similarly among telestroke and control hospitals. Among telestroke hospitals, the mean stroke volume from within the catchment area decreased from 43.6 to 40.6 (change, –3.0 per year) and among control hospitals the mean stroke volume from within the catchment area decreased from 43.0 to 40.3 (change, –2.7 per year), with an estimated differential change of –0.13 (*P* = .97) (Table 2).

**Table 2.** Differential Changes in Hospital-Level Adjusted Outcomes for Telestroke and Control Hospitals^a^

Outcomes	2 y Before implementation	2 y After implementation	Change	Difference-in-differences	*P* value for difference
Annual stroke volume per hospital, mean (SD), No. of patients					
Telestroke	79.6 (58.2)	76.3 (57.3)	–3.3	0.009	>.99
Control	78.8 (67.1)	75.5 (66.7)	–3.3		
Annual stroke volume per hospital from within catchment, mean (SD), No. of patients					
Telestroke	43.6 (43.6)	40.6 (42.4)	–3.0	–0.13	.97
Control	43.0 (50.6)	40.3 (49.9)	–2.7		
Distance traveled per patient by ambulance, mean (SD), km^b^					
Telestroke	13.97 (16.8)	14.15 (16.8)	0.18	–0.27	.36
Control	14.03 (16.8)	14.55 (16.8)	0.52		
Patient case mix as captured by estimated 180-d mortality, mean (SD), %					
Telestroke	17.4 (5.0)	17.4 (5.0)	0.0	–0.11	.23
Control	17.3 (5.0)	17.4 (5.0)	0.1		
Proportion of patients transferred, mean (SD), %					
Telestroke	5.7 (23.2)	6.8 (23.2)	1.1	0.17	.48
Control	4.8 (23.2)	5.8 (23.2)	1.0		
Receiving hospital bed count among transferred patients, mean (SD), No.					
Telestroke	646.8 (362.5)	650.2 (358.6)	3.4	6.28	.66
Control	584.8 (358.2)	583.5 (358.4)	–1.3		
Discharging hospital bed count among all patients, mean (SD), No.					
Telestroke	304.9 (256.5)	317.5 (256.5)	12.6	2.01	.43
Control	300.8 (256.4)	311.2 (256.4)	10.4		

^a^ A total of 593 hospital pairs.

^b^ The outcome of miles traveled by ambulance was measured among a 20% random sample of the study population based on data availability.

Among the 20% random sample for whom we had ambulance data, the mean distance traveled by patients arriving by ambulance increased only slightly, and the change was similar between telestroke and control hospitals. Among patients transported to telestroke hospitals, the mean distance traveled by ambulance increased from 14.0 km to 14.2 km from preimplementation to postimplementation (difference, 0.2 km); among patients transported to control hospitals, the mean distance traveled by ambulance increased from 14.0 km to 14.6 km from preimplementation to postimplementation (difference, 0.6 km). The adjusted differential change was not significant (0.4 km; *P* = .36). The patient case mix as reflected by estimated 180-day mortality rates was available for the full sample and was stable from before to after telestroke implementation and were similar in telestroke and control hospitals. Among telestroke hospitals, the mean estimated mortality rate was 17.4% both before and after implementation (change, 0.0) and among control hospitals, the mean estimated mortality rate increased from 17.3% to 17.4% (change, 0.1%) from preimplementation to postimplementation. The adjusted differential change of –0.11 was not significant (*P* = .23).

The proportion of patients with stroke who transferred to another hospital increased similarly from before to after telestroke implementation among both telestroke and control hospitals (from 5.7% to 6.8% among telestroke hospitals and from 4.8% to 5.8% among control hospitals; adjusted difference-in-differences, 0.2%; *P* = .48). Among transferred patients, there was not a significant difference between groups in the change in mean bed size of the receiving hospital (from 646.8 to 650.2 among telestroke hospitals and from 584.8 to 583.5 among control hospitals; adjusted difference-in-differences, 6.3; *P* = .66). Finally, among all patients, the discharging hospital mean bed count increased similarly from preimplementation to postimplementation regardless of presentation to a telestroke or control hospital (from 304.9 to 317.5 among telestroke hospitals and from 300.8 to 311.2 among control hospitals; adjusted difference-in-differences, 2.0; *P* = .43).

### Sensitivity Analyses

We conducted subanalyses separately among PSC hospitals and among non-PSC hospitals. Overall, our findings were not appreciably different; however, we did note that, among PSCs implementing telestroke, there was a differential increase in receiving hospital mean bed count relative to controls from preimplementation to postimplementation (from 650.1 to 651.4 among telestroke hospitals and from 678.6 to 630.8 among control hospitals; adjusted difference-in-differences, 51.0; *P* = .04). This differential change was not found among nonstroke center hospitals (Table 3).

**Table 3.** Differential Change in Adjusted Hospital-Level Outcomes for Telestroke and Control Hospitals, Stratified by Stroke Center Status

Outcome	Primary stroke centers (179 hospital pairs)						Nonstroke center hospitals (414 hospital pairs)				
	2 y Before implementation	2 y After implementation	Change	Difference-in-differences	*P* value for difference	2 y Before implementation		2 y After implementation	Change	Difference-in-differences	*P* value for difference
Annual stroke volume per hospital, mean (SD), No. of patients											
Telestroke	123.3 (73.1)	119.3 (72.0)	–4.0	–0.4	.96	60.6 (47.2)		57.6 (46.5)	–3.0	–0.2	.93
Control	135.4 (86.9)	131.8 (86.7)	–3.6			54.4 (50.1)		51.2 (50.2)	–3.2		
Annual stroke volume per hospital from within catchment, mean (SD), No. of patients											
Telestroke	58.5 (54.0)	55.5 (52.7)	–3.1	0.1	.98	37.2 (36.4)		34.2 (35.3)	–2.9	0.02	.98
Control	64.7 (65.2)	61.6 (64.1)	–3.1			33.6 (39.4)		31.1 (39.0)	–2.5		
Distance traveled per patient by ambulance, mean (SD), km^a^											
Telestroke	12.2 (13.2)	12.7 (13.2)	0.32	0.2	.52	15.3 (16.8)		15.3 (16.8)	0.0	–0.8	.07
Control	12.9 (13.2)	12.9 (13.2)	0.06			15.0 (16.8)		16.3 (16.8)	1.3		
180-d Estimated mortality, mean (SD), %											
Telestroke	17.7 (5.0)	17.6 (5.0)	–0.1	–0.2	.18	17.2 (5.0)		17.2 (5.0)	–0.0	–0.1	.62
Control	17.6 (5.0)	17.6 (5.0)	0.03			17.2 (5.0)		17.3 (5.0)	0.1		
Proportion of patients transferred, mean (SD), %											
Telestroke	3.2 (17.7)	4.1 (17.7)	0.9	0.2	.48	7.4 (26.5)		8.7 (26.5)	1.3	0.1	.83
Control	2.6 (17.7)	3.2 (17.7)	0.7			6.7 (26.5)		7.9 (26.5)	1.2		
Receiving hospital bed count among transferred patients, mean (SD), No.											
Telestroke	650.1 (341.3)	651.4 (341.4)	1.2	51.0	.04	645.5 (362.4)		649.7 (362.6)	4.2	–6.6	.69
Control	678.6 (341.0)	630.8 (341.6)	–47.8			555.1 (362.3)		567.4 (362.3)	12.3		
Discharging hospital bed count among all patients, mean (SD), No.											
Telestroke	345.7 (221.1)	351.4 (221.1)	5.6	3.7	.32	276.2 (276.6)		293.6 (276.7)	17.4	0.1	.98
Control	345.6 (221.1)	347.2 (221.1)	1.6			263.6 (276.6)		280.9 (276.6)	17.3		

^a^ The outcome of distance traveled by ambulance was measured among a 20% random sample of the study population based on data availability.

In another sensitivity analysis, we limited control hospitals to those indicating in a survey that they did not have telestroke. After rematching and repeating the difference-in-differences analysis, our findings again did not change appreciably (eTables 2 and 3 in the Supplement).

## Discussion

In an analysis of 593 US hospitals adopting telestroke from 2009 to 2016, we found no clinically important differential changes in a series of measures of stroke systems of care, including annual volume of patients with stroke arriving by ambulance, hospital case mix, and proportion of patients transferred. Our findings contrast with previous reports from smaller and single-network analyses showing an association between telestroke adoption and a decrease in the proportion of patients transferred and an increased likelihood of remaining in one’s own local community for care.^28,29,30,31^ Our findings may differ for several reasons. First, prior studies were smaller in nature and may not have been generalizable. Second, some studies used designs that did not fully address temporal trends. Third, publication bias is possible, with positive outcomes more likely to be published. Fourth, most studies were prior to 2015 and the establishment of benefit associated with endovascular thrombectomy. Evolving data for endovascular thrombectomy may change decision-making regarding the need for advanced imaging and evaluation of eligibility, and telestroke may facilitate identification of more patients who may experience a benefit from transferring to another hospital.^32,33^

Among transferred patients, the change in the size of the receiving hospital from preimplementation to postimplementation of telestroke was minimal and similar between telestroke and control hospitals overall. However, among patients transferred from PSCs, those from telestroke hospitals were transferred to larger hospitals after implementation relative to those transferred from control PSCs. Implementation of telestroke at PSCs may be associated with changes in the approach to transferred patients or differential recognition of the need for resources of larger hospitals.

We were surprised by the lack of change in prehospital triage. Our expectation was that the implementation of telestroke would be associated with EMS professionals preferentially choosing newly capable telestroke hospitals and that this would be reflected in decreased ambulance transport distances and increased stroke volume. It is possible that EMS protocols or professionals may not consider telestroke equivalent to in-person neurology or may have other reasons for not redirecting patients. Alternatively, it is also possible that the implementation of telestroke would, in fact, be associated with patient and EMS decision-making, but these stakeholders are not aware of the new capabilities. We also did not consider how often telestroke was used at the hospitals. It is possible that some telestroke hospitals did not use this service frequently, and therefore, there was no substantive change in care patterns.^34^

Our results do not imply that telestroke is not helpful. Telestroke is clearly associated with improved quality of care and outcomes for individual patients with stroke,^35,36,37,38^ and many ED physicians value the ability to consult stroke neurologists.^39^ Telestroke may also enable a hospital to attain stroke center certification. However, our results suggest that, to date, telestroke has not been significantly associated with patient destination or transfer patterns.

Our results also do not imply that telestroke is not associated with outcomes in stroke systems of care. It may be that meeting this goal requires more deliberate changes in prehospital transport or system protocols. More work is required to assess what changes are needed in stroke systems of care—whether through more specific EMS protocols to ensure optimal prehospital patient sorting or more collaborative hospital networks to optimize interhospital sorting with respect to transfer and final destinations.

### Limitations

Our study has several limitations. We included patients with stroke and TIA. We chose to include patients with TIA so we could comprehensively assess the association of telestroke with outcomes; however, this inclusion may have biased our results toward the null. Our definition of catchment area led to the exclusion of 9% of telestroke hospitals. We thought that this definition was the clearest to interpret. However, it is possible that this definition introduced bias associated with our included sample or biased our results toward the null if we were unable to capture increases in stroke volume by this strategy. We identified stroke centers that were certified by national agencies; if any state-designated centers were not also certified nationally, this could have resulted in misclassification. Our measure of hospital stroke case mix does not include a measure of stroke severity, which makes evaluation of the likely proportion of endovascular thrombectomy–eligible patients among those transferred impossible to assess. It is also possible that the 2-year postimplementation period was too short to capture resulting changes. Finally, it is possible that hospitals in our control group had telestroke services, which would bias our results toward the null. The similar findings in our sensitivity analysis using self-reported telestroke status in a survey is reassuring, but this analysis was limited to non-PSCs.

## Conclusions

In a national study of hospitals with telestroke services, we found that the introduction of telestroke was not associated with any substantive changes in the outcomes of patients with stroke or in the stroke systems of care we could capture, including hospital patient volume and transfer proportions. If telestroke is implemented with the goal of improving stroke systems of care, additional policy-level interventions may be required to meet that goal.

## Notes

Supplement. **eMethods 1.** Details of 180-Day Predicted Mortality Model **eMethods 2.** Specifications of the Difference-in-Differences Model **eMethods 3.** Differences in 2 Years Pre-Implementation Trends in Outcomes **eTable 1.** Assessment of Parallel Trends Assumption **eTable 2.** Comparison of Telestroke and Control Hospitals After Matching (Limited to Control Hospitals Confirmed via National Emergency Department Inventory) **eTable 3.** Differential Changes in Adjusted Outcomes for Telestroke and Control Hospitals (Limited to Control Hospitals Confirmed via National ED Inventory; 353 Hospital Pairs)
