# Error in Byline

**DOI:** 10.1001/jamanetworkopen.2021.35869

**Published:** 2021-10-26

In the Original Investigation titled “Association of Hospital Telestroke Adoption With Changes in Initial Hospital Presentation and Transfers Among Patients With Stroke and Transient Ischemic Attacks,”^1^ published September 23, 2021, the fourth author’s name should be Zubizarreta, not Zubizaretta. This article has been corrected.^1^
