# Supplementary material for: Association of Hospital Telestroke Adoption With Changes in Initial Hospital Presentation and Transfers Among Patients With Stroke and Transient Ischemic Attacks
Source: JAMA Netw Open. 2021 Sep 23;4(9):e2126612. doi: 10.1001/jamanetworkopen.2021.26612 (PMC8461501; doi:10.1001/jamanetworkopen.2021.26612)
Supplement: Supplement. — eMethods 1. Details of 180-Day Predicted Mortality Model eMethods 2. Specifications of the Difference-in-Differences Model eMethods 3. Differences in 2 Years Pre-Implementation Trends in Outcomes eTable 1. Assessment of Parallel Trends Assumption eTable 2. Comparison of Telestroke and Control Hospitals After Matching (Limited to Control Hospitals Confirmed via National Emergency Department Inventory) eTable 3. Differential Changes in Adjusted Outcomes for Telestroke and Control Hospitals (Limited to Control Hospitals Confirmed via National ED Inventory; 353 Hospital Pairs) [file jamanetwopen-e2126612-s001.pdf]

Supplementary Online Content

Zachrison KS, Richard JV, Wilcock A, et al. Association of hospital telestroke adoption with changes in initial hospital presentation and transfers among patients with stroke and transient ischemic attacks. *JAMA Netw Open.* 2021;4(9):e2126612. doi:10.1001/jamanetworkopen.2021.26612

eMethods 1. Details of 180-Day Predicted Mortality Model

eMethods 2. Specifications of the Difference-in-Differences Model

eMethods 3. Differences in 2 Years Preimplementation Trends in Outcomes

eTable 1. Assessment of Parallel Trends Assumption

eTable 2. Comparison of Telestroke and Control Hospitals After Matching (Limited to Control Hospitals Confirmed via National Emergency Department Inventory)

eTable 3. Differential Changes in Adjusted Outcomes for Telestroke and Control Hospitals (Limited to Control Hospitals Confirmed via National ED Inventory; 353 Hospital Pairs)

This supplementary material has been provided by the authors to give readers additional information about their work.

© 2021 Zachrison KS et al. *JAMA Network Open.*

eMethods 1. Details of 180-Day Predicted Mortality Model

To capture case severity at individual hospitals we measured each patient's predicted 180-day mortality from admission. Building on our prior work, this was based on patient demographics, enrollment in Medicaid in the prior 12 months, original entitlement reason, inpatient and postacute care facility use in the prior 12 months, and chronic disease burden (based on 27 disease indicators) prior to admission: dementia, alzheimers, acute myocardial infarction, asthma, atrial fibrillation, cataract, congestive heart failure, chronic kidney disease, endometrial cancer, breast cancer, colon cancer, lung cancer, prostate cancer, chronic obstructive pulmonary disease, depression, diabetes mellitus, glaucoma, history of hip fracture, hyperlipidemia, benign prostatic hyperplasia, tension-type headache, thyroid disease, history of ischemic heart disease, osteoporosis, arthritis, and prior stroke.

© 2021 Zachrison KS et al. *JAMA Network Open.*

eMethods 2. Specifications of the Difference-in-Differences Model

We used a generalized linear model for each outcome of interest for hospital *h* at time *t* as follows:

$$Y_{ht} = \beta_0 + \beta_1 T_h + \beta_2 P_t + \beta_3 T_h * P_t + \beta_4 X_h + \varepsilon$$

Where Y_{ht} is the outcome for hospital h at time t; T_h is an indicator variable for the telestroke status of the hospital (control: 0, telestroke: 1); P_t gives the time period (pretelestroke vs. posttelestroke implementation), and X_h are other covariates in the model listed below. In all models we accounted for clustering by matched treatment and control hospital pairs. The primary effect of interest in the difference-in-differences approach is given by β_3 which captures whether the relationship between time (preimplementation vs. postimplementation) and the outcome varies by telestroke status.[26]

© 2021 Zachrison KS et al. *JAMA Network Open.*

eMethods 3. Differences in 2 Years Preimplementation Trends in Outcomes

A key test in a difference-in-difference analysis is to compare trends in outcomes before the intervention. This is one test of the underlying assumption that the control hospitals represent the counterfactual to the trends that would have been seen if the intervention had not been implemented. The key question is whether the trends were parallel.

To estimate pre-period trends in our outcomes, we used the 4 consecutive 6-month periods of preimplementation data for each paired telestroke and control hospital. Among telestroke and control hospitals we used generalized linear models for each outcome, and tested an interaction, with models specified as follows for hospital *h* at time *t* as follows:

$$Y_{ht} = \beta_0 + \beta_1 T_h + \beta_2 T_h * P_t + \beta_3 X_h + \varepsilon$$

Where Y_{ht} is the outcome for hospital h at time t; T_h is an indicator variable for the telestroke status of the hospital (control: 0, telestroke: 1); P_t gives the time period (by 6-month block), and X_h are other covariates in the model.

eTable 1 gives the parameter estimates and significance for the interaction term assessing parallel trends.

© 2021 Zachrison KS et al. *JAMA Network Open.*

eTable 1. Assessment of Parallel Trends Assumption

Outcome	2 Year Preintervention Period Trend Estimate vs. Control	*P* Value
Stroke volume per hospital	51.45	0.86
Stroke volume from catchment area	22.1	0.96
Distance travelled by ambulance	-0.004	0.99
180-day predicted mortality	-0.03%	0.53
Proportion of patients transferred	0.17%	0.10
Bed count of receiving hospital	-2.28	0.75
Bed count of discharging hospital	-0.04	0.97

© 2021 Zachrison KS et al. *JAMA Network Open.*

eTable 2. Comparison of Telestroke and Control Hospitals After Matching (Limited to Control Hospitals Confirmed via National Emergency Department Inventory)

	Telestroke Hospitals (n = 353)		NEDI Control Hospitals (n = 353)	
	n	%	n	%
Primary Stroke Center*				
Yes	NA	NA	NA	NA
No	NA	NA	NA	NA
Urban vs. Rural*				
Urban	222	62.9%	222	62.9%
Rural	131	37.1%	131	37.1%
Number of Beds* (Terciles)				
<30 (T1)	122	34.6%	122	34.6%
30-142 (T2)	146	41.4%	146	41.4%
143+ (T3)	85	24.1%	85	24.1%
Hospital Alternatives* (Terciles)				
Fewest options (T1)	130	36.8%	130	36.8%
Few options (T2)	125	35.4%	125	35.4%
More options (T3)	98	27.8%	98	27.8%
Hospital Type*				
STACH	227	64.3%	227	64.3%
CAH	126	35.7%	126	35.7%
Census Region of Hospital				
1 - Northeast	34	9.6%	37	10.5%
2 - Midwest	101	28.6%	104	29.5%
3 - South	137	38.8%	133	37.7%
4 - West	81	23.0%	79	22.4%
Ownership				
Public	98	27.8%	94	26.6%
Private	255	72.2%	259	73.4%
Annual Stroke Volume, 2008 (Terciles)				
T1 (<30)	110	31.2%	111	31.4%
T2 (30-76)	147	41.6%	151	42.8%
T3 (77+)	96	27.2%	91	25.8%

Legend. T1: tercile 1; T2: tercile 2; T3: tercile 3; STACH: short term acute care hospital; CAH: critical access hospital; NEDI: National Emergency Department Inventory

© 2021 Zachrison KS et al. *JAMA Network Open.*

eTable 3. Differential Changes in Adjusted Outcomes for Telestroke and Control Hospitals (Limited to Control Hospitals Confirmed via National ED Inventory; 353 Hospital Pairs)

**Outcomes**	**2 Years Preimplementation**	**2 Years Postimplementation**	**Change from Pre- to Post-**	**Difference-in-Differences**	***P* Value for difference**
Annual Stroke Volume per Hospital (mean)					
Telestroke	53.8	50.0	-3.7	-0.4	0.84
Control	47.6	44.3	-3.3		
Annual Stroke Volume from Within Catchment (mean per hospital)					
Telestroke	32.4	29.6	-2.8	-0.5	0.72
Control	28.4	26.1	-2.3		
Distance Travelled by Ambulance (mean miles per patient) [a]					
Telestroke	9.57	9.39	-0.2	-1.2	0.04
Control	8.53	9.58	1.0		
180-day predicted mortality (mean)					
Telestroke	17.3%	17.3%	0.0%	-0.0%	0.64
Control	17.3%	17.3%	0.0%		
Proportion of patients transferred					
Telestroke	7.9%	9.3%	1.3%	-0.2%	0.55
Control	6.9%	8.4%	1.6%		
Receiving Hospital Bed Count among transferred patients (mean)					
Telestroke	648.7	654.4	5.6	9.5	0.60
Control	573.9	572.2	-1.7		
Discharging Hospital Bed Count among all patients (mean)					
Telestroke	279.9	298.8	18.9	0.9	0.85
Control	263.7	281.6	17.9		

Legend. [a]The outcome of miles travelled by ambulance was measured among a 20% random sample of the study population due to data availability.

© 2021 Zachrison KS et al. *JAMA Network Open.*
